# Structure, Activity Regulation, and Role of the Mitochondrial Calcium Uniporter in Health and Disease

**DOI:** 10.3389/fonc.2017.00139

**Published:** 2017-07-10

**Authors:** Cristina Mammucari, Gaia Gherardi, Rosario Rizzuto

**Affiliations:** ^1^Department of Biomedical Sciences, University of Padova, Padova, Italy

**Keywords:** mitochondrial calcium uniporter, mitochondrial calcium uniporter pathophysiology, mouse models, breast cancer, mitochondrial calcium uniporter regulators

## Abstract

Mitochondrial Ca^2+^ uptake plays a pivotal role both in cell energy balance and in cell fate determination. Studies on the role of mitochondrial Ca^2+^ signaling in pathophysiology have been favored by the identification of the genes encoding the mitochondrial calcium uniporter (MCU) and its regulatory subunits. Thus, research carried on in the last years on one hand has determined the structure of the MCU complex and its regulation, on the other has uncovered the consequences of dysregulated mitochondrial Ca^2+^ signaling in cell and tissue homeostasis. Whether mitochondrial Ca^2+^ uptake can be exploited as a weapon to counteract cancer progression is debated. In this review, we summarize recent research on the molecular structure of the MCU, the regulatory mechanisms that control its activity and its relevance in pathophysiology, focusing in particular on its role in cancer progression.

## Introduction

The role of mitochondria in cancer was long debated. In the 20s of the last century, Otto Warburg made the pivotal observation that cancer cells mostly rely on glycolysis for ATP production even in the presence of optimal pO_2_. Warburg proposed that mitochondrial dysfunction was the primary cause of preferential aerobic glycolysis. In addition, only cells able to increase glycolytic ATP production during periods of respiratory failure would progress toward cancer. Thus, cancer would be a consequence of progressive and irreversible defect in oxidative metabolism. However, experimental evidence revealed the weaknesses of this model. First, in some tumors, cells have normal respiratory capacity and/or are able to reactivate oxidative phosphorylation in case of impaired glycolysis. Second, in alternative to glucose metabolism, certain cancers utilize glutamine as an energy source (1). In addition, Warburg’s theory did not explain the existence of cancer-related gene mutations and, finally, it was unclear how impaired respiration would lead to uncontrolled cell growth. As a result, for many years, tumor metabolism was overlooked, in favor of a more accepted view of cancer as genetic disease. Recently, the importance of metabolism in cancer progression has been reconsidered. Mutations in genes encoding mitochondrial proteins and mitochondrial dysfunctions are linked to cancer progression and respiration defects contribute to genome instability. In addition, damage in respiration underlies genome instability which, in turn, sustains respiratory impairment and cancer progression (2). Mitochondrial Ca^2+^ signaling plays different roles in pathophysiology. On one hand, by buffering cytosolic [Ca^2+^], it regulates cell processes controlled by intracellular [Ca^2+^], like those regulated by Ca^2+^-binding proteins. On the other side, within mitochondria, Ca^2+^ regulates three TCA cycle enzymes, thus exerting a positive role on oxidative metabolism. However, excessive mitochondrial Ca^2+^ entry consequent to stress stimuli causes opening of the mitochondrial permeability transition pore (mPTP) and release of proapoptotic factors which eventually lead to cell death (3). On this basis, for a long time, the study of mitochondrial Ca^2+^ signaling in cancer biology has been primarily focused on the Ca^2+^-dependent activation of the proapoptotic axis as a putative therapeutic strategy. However, once stimulated with an appropriate agonist, cancer cells display large mitochondrial Ca^2+^ peaks, raising the question whether effective mitochondrial Ca^2+^ accumulation might play a permissive role in cancer progression. Thus, the scenario is quite complex, and recent literature suggests that mitochondrial Ca^2+^ homeostasis plays different roles in cancer, depending on the type and stage of the tumor and on whether primary tumors or metastasis are being analyzed.

Once the electrophysiological properties of the mitochondrial calcium uniporter (MCU), the highly selective channel responsible for mitochondrial Ca^2+^ uptake, were described (4), the genes encoding both the pore and the regulatory subunits have been eventually identified (3). Therefore, studies aimed at further delving into the role of mitochondrial Ca^2+^ homeostasis in pathophysiology, including cancer progression, were conducted based on the molecular modulation of the MCU complex components.

Genetic modulation of MCU expression confirmed the previously recognized role of mitochondrial Ca^2+^ overload in triggering cell death. Thus, the old paradigm of excessive Ca^2+^ uptake being permissive for apoptotic cell death still holds true. However, other studies highlighted novel and still incompletely defined aspects of MCU in cancer, indicating that mitochondrial Ca^2+^ uptake plays multiple and complex roles in cancer biology. This review will summarize the molecular structure of MCU, the regulatory mechanisms underlying its activity, and will uncover its role in pathophysiology, with particular attention to cancer biology.

## Ca^2+^ Transport through the Outer Mitochondrial Membrane

Voltage-dependent anion channels (VDACs) are the most abundant proteins of the outer mitochondrial membrane. VDAC1, VDAC2, and VDAC3 are permeable to <5 kDa metabolites and are characterized by a weak anion selectivity in the open channel conformation, while they show strong cation preference in the closed states (5). The three VDAC isoforms share comparable channel properties in Ca^2+^ transport from the cytosol to the intermembrane space (6); however, they differ in specific functions. In particular, VDAC1, by interacting with the InsP3R located at the ER, specifically senses low amplitude proapoptotic Ca^2+^ signals (7). In addition, isoform-specific interactions of VDAC with apoptotic regulators contribute to the selective control of cell fate (3). VDACs are involved in many other functions, including metabolism regulation and energy production. For further discussion on VDACs, the reader is referred to specialized publications (8–11).

## MCU: Molecular Structure and Activity Regulation

**Figure 1:**
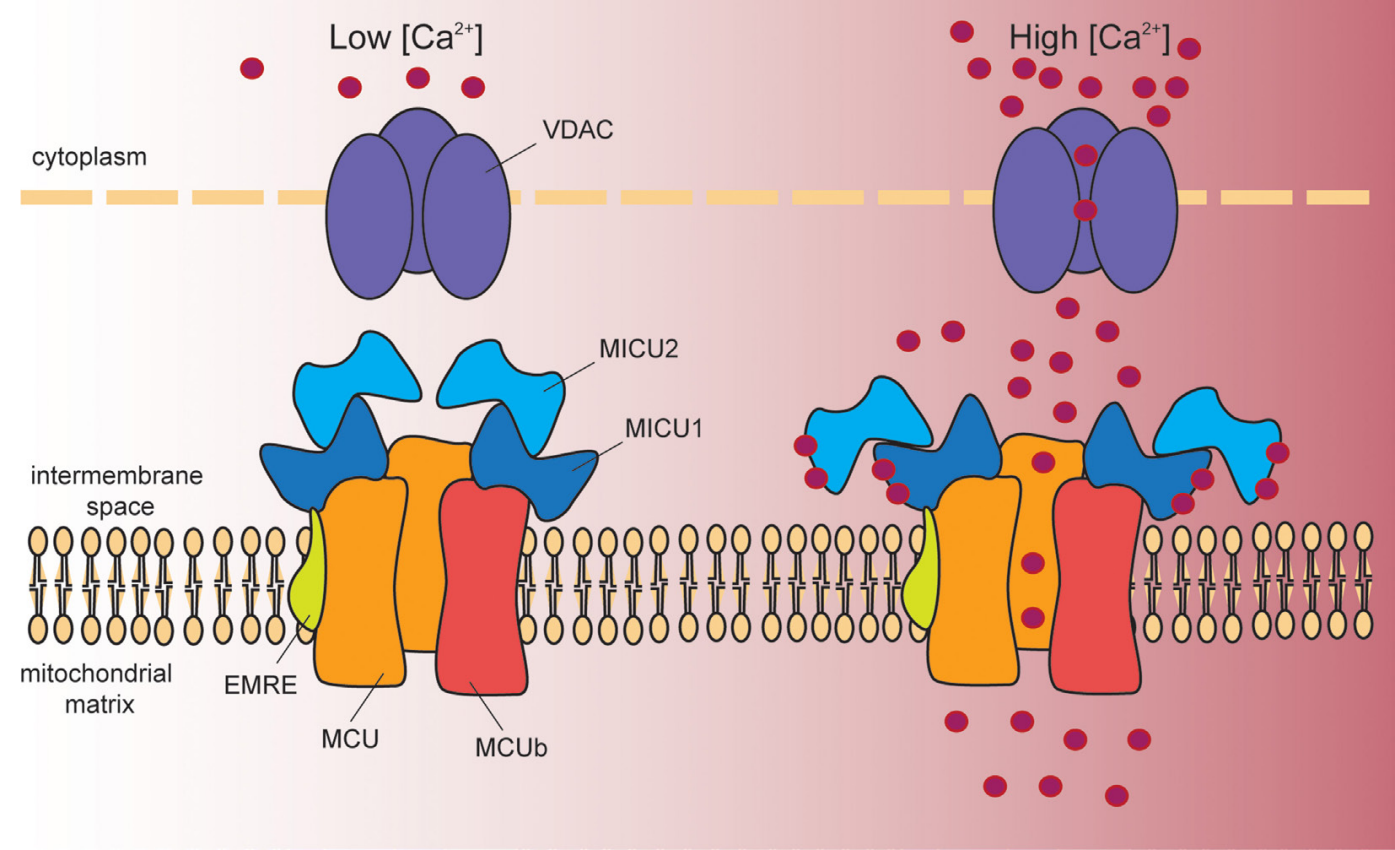
Schematic representation of the proteins involved in mitochondrial Ca^2+^ entry. Cytosolic Ca^2+^ enters into the mitochondrial matrix according to the mitochondrial membrane potential (−180 mV, negative inside). At the outer mitochondrial membrane, voltage-dependent anion channels (VDAC1–3) ensure permeability to solutes smaller than 5 kDa, including Ca^2+^. At the inner mitochondrial membrane, a multiprotein complex controls mitochondrial Ca^2+^ uptake. This complex is composed of the pore-forming subunits MCU and MCUb, and by the regulatory proteins MICU1, MICU2, and EMRE. In resting conditions (left hand side), MICU1/MICU2 heterodimers act as MCU gatekeeper, due to the inhibitory effect of MICU2. Once Ca^2+^ signaling is activated (right hand side), the increase in cytosolic [Ca^2+^] induces a conformational change in the dimer that releases MICU2-dependent inhibition. At the same time, MICU1 acts as a cooperative activator of the channel, and thus stimulates channel activity. EMRE stabilizes MCU–MICU1 complex contributing to fine-tuned Ca^2+^ entry into mitochondria.

A 480 kDa inner mitochondrial membrane (IMM) multimeric complex, i.e., the MCU, comprising channel pore-forming subunits and regulatory elements, ensures Ca^2+^ entry into mitochondria (Figure 1). Long before the electrophysiological properties and the molecular structure of MCU were disclosed, cell Ca^2+^ signaling was already extensively characterized. The driving force for Ca^2+^ entry into mitochondria is represented by the electrochemical gradient across the IMM, which is negative in the mitochondrial matrix (about −180 mV). However, despite this huge driving force, resting mitochondrial [Ca^2+^] is in the nanomolar range, indicating that the affinity of the channel for Ca^2+^ is low. In addition, the mitochondrial Na^+^/Ca^2+^ exchanger ensures efficient extrusion of Ca^2+^ from mitochondria (12). Upon stimulation with InsP3-generating agonists, cytosolic Ca^2+^ transients reach the micromolar range and are paralleled by sharp mitochondrial [Ca^2+^] increases. This apparent conundrum is explained by the fact that ER and mitochondria share sites of near proximity, where the distance between the two organelles is extremely close (13, 14). These sites are named mitochondrial associated membranes (MAMs). At MAMs, Ca^2+^ released from the ER generates microdomains of high [Ca^2+^], which are sensed by mitochondria and which are responsible for the significant increase in mitochondrial [Ca^2+^]. The amount of Ca^2+^ uptake by mitochondria follows a non-linear relationship related to the amount of Ca^2+^ present at microdomains. Ca^2+^ uptake is inefficient not only at low [Ca^2+^], for the reasons explained above, and for the newly described role of MCU modulators (see below), but also in the presence of too high [Ca^2+^] at which the inhibitory effect of Ca^2+^ on InsP3R prevails. Thus, efficient mitochondrial Ca^2+^ uptake is warranted by the prompt removal of Ca^2+^ from microdomains exerted by mitochondria.

In 2004, Clapham and colleagues, by patch-clamping mitoplasts preparations, unequivocally identified a Ca^2+^-selective mitochondrial channel whose current had the properties of that mediated by MCU (4). Subsequent characterization demonstrated that different tissues display different MCU activity, according to the specific physiological properties of Ca^2+^ signaling in each tissue, suggesting that tissue-specific regulation of the channel occurs (15).

### MCU

More recently, the Mootha and Rizzuto groups, by different methods, in parallel achieved the identification of *ccdc109a* as the gene encoding the pore-forming subunit of MCU, sufficient *per se* to ensure channel activity (16, 17). MCU was co-expressed with the previously described regulatory subunit MICU1 (18), it was required for mitochondrial Ca^2+^ uptake and, most importantly, the purified protein reconstituted in planar lipid bilayers recapitulated the known electrophysiological properties of MCU. Structurally, MCU protein contains two coil-coiled domains and two transmembrane (TM) domains separated by a short hydrophilic loop enriched in acidic amino acids (16, 17). Electron microscopy of mitochondria overexpressing tagged MCU demonstrated that both the N- and the C-terminus of MCU face the matrix (19). By combining structural bioinformatics techniques and molecular dynamic simulations, a quaternary structure was proposed, and tridimensional localization of critical residues of the pore was modeled (20). Each pore-forming subunit consists of two helical membrane spanning domains connected by a short loop containing a “DIME” motif. This region contains negatively charged residues which have been previously identified to play an essential role in MCU-mediated Ca^2+^ uptake (16, 17). These residues are part of a narrow selectivity filter which accommodates a single Ca^2+^ ion. One additional feature is represented by the C-terminal tails, which define a pore of 1 Å of diameter, suggesting that this MCU model stably adopts a close conformation, as it commonly occurs for Ca^2+^ channels (20). Subsequently, a crystal structure at a 1.80 Å resolution of the MCU N-terminal domain (NTD) was released (21). This analysis identified one α-helix and six β-strands that form the central core, two highly conserved loops and one leucine-rich short α-helix, which stabilizes the hydrophobic interior of MCU NTD. One additional α-helix and a C-terminal tail are also present. MCU NTD is essential for regulation of Ca^2+^ uptake, either through interaction with regulators, or by post-transcriptional modifications. Finally, the structure of the pore domain of MCU from *Caenorhabditis elegans* was determined by nuclear magnetic resonance (NMR) and electron microscopy. Differently from the quaternary complex proposed for mammalian MCU (20), a pentameric organization was proposed (22), in which the TM domain consists of 10 helices arranged in two concentric layers. Specific aspartate and glutamate residues were identified as forming the selectivity filter. The proposed structure is completely different from other Ca^2+^ channels and leaves many open questions concerning Ca^2+^ binding and transport, and regulation of MCU conformation by its interactors. Further details were recently added by an NMR-based analysis aimed at better characterizing the properties of the selectivity filter. This analysis conclusively demonstrated that the double carboxylate rings formed by the “DXXE” motif is the ion selectivity filter and that cooperativity of the two ion binding sites confers high ion affinity (23).

Genetic manipulation of MCU in animal models gave interesting results. In lower organisms, i.e., *Trypanosoma brucei* and *Danio rerio*, MCU knockdown or knockout caused developmental defects. In the parasite, MCU silencing or conditional knockout impaired mitochondrial Ca^2+^ uptake as expected, reduced energy production and increased AMP/ATP ratio and autophagosome formation. As a result, growth was markedly inhibited as well as infectivity in mice (24). In zebrafish, MCU silencing caused major developmental defects, impinging on RhoA signaling and F-actin dynamics (25). In *Drosophila melanogaster*, overexpression of a dominant-negative isoform of MCU (DN-MCU) in mushroom body neurons during pupation caused memory impairment associated with a decrease in synaptic vesicles and an increased length in the axons (26), again highlighting the importance of efficient mitochondrial Ca^2+^ uptake for development. On the contrary, *C. elegans* MCU null mutants are viable and fertile. Studies performed in the worm help elucidating the involvement of mitochondrial Ca^2+^ uptake and ROS production in the wound repair mechanism, demonstrating that wounding is paralleled by an increase in mitochondrial [Ca^2+^] and ROS production. ROS are required for proper repair, and MCU deletion causes impaired wound healing (27). Deletion of MCU in the mouse has opposite developmental effects depending on the strain. MCU^−/−^ embryos in the C57BL/6 background die around E 11.5–E 13.5, for causes that are not completely clear (28, 29). Viable mice were instead obtained in the outbred CD1 background, although not at Mendelian ratio (30). In the latter case, defects in MCU^−/−^ mice were mostly apparent in the diminished performance of skeletal muscle. MCU^−/−^ hearts were as susceptible as wild-type hearts to ischemia/reperfusion (I/R) injury, although a difference was apparent in the effects of the mPTP inhibitor cyclosporin A (CsA). In contrast to wild-type hearts, MCU^−/−^ hearts were not protected from I/R injury by CsA treatment. These data indicate that, besides mPTP opening by mitochondrial Ca^2+^ overload, alternative damaging pathways are activate by I/R which are not counteracted by MCU deletion (30). One of the more obvious and more explored interpretation for the unexpected lack of marked phenotype is that MCU^−/−^ animals could have developed compensatory mechanisms that allow them to cope with developmental challenges and with stresses encountered during postnatal life.

Comparison of MCU activity in patch-clamped mitoplasts from different tissues demonstrated great variability (15). In particular, heart is characterized by very low MCU current density, which is 30-fold higher in skeletal muscle. However, the biophysical properties of heart and skeletal muscle MCU currents are identical, indicating that different tissues share the same Ca^2+^ channel and that tissue-specific regulatory mechanisms are responsible for the differences in channel activity. These differences are explained by the fact that MCU is part of a large protein complex, comprising both other channel-forming subunits and regulatory components, as detailed hereafter.

### MCUb

A closely related protein with inhibitory activity on channel function is encoded by the *ccdc109b* gene and is named MCUb (20). MCUb shares 50% similarity with MCU and, like MCU, it contains two TM domains. However, once inserted in lipid bilayers, it is not able to conduct any current and, co-expressed with MCU, completely abolishes MCU currents, thus exerting a dominant-negative effect on the channel activity. MCU forms both homo-oligomers and hetero-oligomers with MCUb. However, MCUb is expressed at different levels compared to MCU in different tissues, indicating that it plays a tissue-dependent modulatory role.

### EMRE

By quantitative mass spectrometry of the uniporter complex, a 10 kDa protein containing a single TM domain was purified (31). This protein was named EMRE (essential MCU regulator), since its abrogation depleted uniporter channel activity and interaction of MCU with MICU1 and MICU2 (which are described below). However, whether EMRE is really essential for uniporter activity has been questioned, since organisms like plants and fungi, in which MCU and MICU1 are highly conserved, do not express EMRE. In addition, mice deleted for both EMRE alleles had normal body weight and no signs of ataxia or skeletal muscle defects (32). To clarify this point, the yeast *Saccharomyces cerevisiae*, which lacks MCU, was used as *in vivo* system to reconstitute uniporter activity. While expression of *Dictyostelium discoideum* MCU alone was sufficient to reconstitute uniporter activity, the human homolog required co-expression of EMRE (33). This observation clarifies the difference in uniporter component expression among different species and sheds light on the evolution of the MCU. According to recent data, EMRE not only keeps in place MCU and MICUs, but actively plays a regulatory role on channel function. Specifically, one report suggested that EMRE, in complex with MCU and MICUs, spans the inner membrane and acts as a sensor of [Ca^2+^] at both sides. By sensing matrix [Ca^2+^] it inhibits MCU activity. EMRE mutants depleted of acidic residues facing the matrix loose this function, resulting in MCU activation and rising of matrix [Ca^2+^]. Thus, in this configuration, EMRE contributes to mitochondrial Ca^2+^ uptake avoiding mitochondrial Ca^2+^ overload (34). Studies performed with mouse uniporter proteins reconstituted in yeast proposed an alternative model. In contrast to the previously proposed topology with the C-terminus facing the mitochondrial matrix (34), in yeast mitochondria, mouse EMRE spans the inner membrane with the NTD facing the matrix, and specific residues located both at the C-terminal and NTDs are required for interaction with MCU. From genetic studies, the authors suggested that the role of EMRE is to keep MCU in an open conformation, thus ensuring Ca^2+^ entry (35). Further details were added by a study performed in HEK 293 cells, in which the topology of EMRE with the N-terminal facing the matrix was confirmed. Mutants in which either the N- or the C-terminal domains were deleted still supported uniporter activity, while substitutions of critical amino acids of the TM helix, which appeared to be essential for interaction with MCU, caused complete loss of channel activity (36). However, in this case, EMRE mutant deleted at the NTD still contained the critical amino acid previously reported as essential for interaction with MCU (35). EMRE was required to ensure that Ca^2+^ entries mitochondria only above a certain cytosolic [Ca^2+^], and this was achieved by binding of the C-terminal tail to MICU1. Thus, EMRE was proposed to act as stabilizer or MCU–MICU1 interaction (36) and assembly of the MCU complex, comprising MCU, EMRE, and regulators of the MICU family, ensures fine-tuned mitochondrial Ca^2+^ entry. Physiological EMRE turnover, ensured by mitochondrial AAA (m-AAA) protease, is essential for correct assembly of the gatekeeping subunits (37, 38). In detail, m-AAA protease degrades the surplus fraction of EMRE that would interact with MCU alone and that would increase the amount of constitutively open channel with consequent Ca^2+^ leakage into mitochondria.

### MICU1

Mitochondrial calcium uniporter channel activity is characterized by a sigmoidal response respective to changes in cytosolic [Ca^2+^]. Little Ca^2+^ enters through the channel at low cytosolic [Ca^2+^]. Upon cell stimulation with an appropriate stimulus, cytosolic [Ca^2+^] increases, followed by rapid and efficient mitochondrial Ca^2+^ entry (3). These properties are determined by the presence of MCU regulators, which exert either a gatekeeping or a stimulatory function on channel activity.

In 2010, the Mootha group applied an integrated approach based on data from comparative physiology, evolutionary genomics, and organelle proteomics, to identified human genes involved in mitochondrial Ca^2+^ entry. Targeted RNAi screen leads to the identification of MICU1, to which an essential role in regulating agonist-induced mitochondrial Ca^2+^ uptake was recognized (18). Subsequent work revealed the complexity of MCU regulation and, in this context, the role of MICU1 was reassessed. MICU1 silencing was reported to increase basal mitochondrial [Ca^2+^], causing mitochondrial Ca^2+^ overload and increasing sensitivity to apoptotic stimuli (39). Accordingly, it was proposed that, by interacting with MCU, MICU1 sets the Ca^2+^ threshold of MCU activation, without affecting the kinetic properties of MCU-mediate Ca^2+^ uptake. Thus, the concept of “gatekeeper” for MICU1 function was introduced. Analysis of MICU1/MCU interaction established that MICU1 localizes in the inner membrane space and forms homo-oligomers. MICU1 domains responsible for interaction with MCU and for gatekeeper function were also identified (40). Further work confirmed the gatekeeping function that MICU1 exerts at low cytosolic [Ca^2+^] but also demonstrated that MICU1 contributes to cooperative activation of mitochondrial Ca^2+^ uptake at high cytosolic [Ca^2+^]. MICU1 plays this dual function by sensing cytosolic [Ca^2+^] at the outer surface of the IMM (41). Crystal structure studies of Ca^2+^-free and Ca^2+^-bound human MICU1 demonstrated that Ca^2+^-free MICU1 forms a hexamer capable of binding to MCU. Upon Ca^2+^ binding, large conformational changes occur within one of the two canonical Ca^2+^ binding EF-hand domains present in the MICU1 sequence. Consequently, disassembly of the hexamer and formation of multiple oligomers of MICU1 dimer occurs after Ca^2+^ binding. The affinity of MICU1 for Ca^2+^ was calculated in the range of 15–20 µM. Thus, at resting cytosolic [Ca^2+^], which is approximately 0.1 µM, MICU1 is free of Ca^2+^ and keeps the MCU channel closed. Upon cell stimulation, cytosolic [Ca^2+^] rises and Ca^2+^ binds to MICU1, inducing MICU1 conformational changes and eventually MCU activation (42). Concerning the pathophysiological function of MICU1, loss-of-function mutations cause a disease phenotype comprising muscle weakness associated with myofiber regeneration, learning difficulties and extra-pyramidal movement disorder. This is due to the fact that loss of MICU1 activity causes an increase in basal mitochondrial [Ca^2+^] and a reduction in cytosolic [Ca^2+^], as demonstrated by studies carried on in patient fibroblasts. The mitochondrial network is fragmented despite maintenance of normal ΔΨ (43). In the available MICU1^−/−^ mouse models, MICU1 deletion causes either complete (44) or significant (32) perinatal mortality. Importantly, the phenotype of surviving MICU1^−/−^ animals mirrors the muscle weakness and neurological defects observed in patients. Similarly to patient fibroblasts, MICU1^−/−^ mice display increased resting mitochondrial Ca^2+^ levels and altered mitochondria morphology (32). Interestingly, in aging animals, resting mitochondrial [Ca^2+^] is no longer significantly different between MICU1^−/−^ and wild-type mice, and phenotypic parameters are also ameliorated, suggesting that MICU1^−/−^ mitochondria undergo a functional remodeling. At the same time, MICU1 deletion causes a marked decrease in EMRE protein levels in mitochondria of young animals, which is even more evident in older mice. Genetic evidence indicates that EMRE heterozygosity rescues the partial lethality and the muscle and brain defects caused by MICU1 deletion. However, the scenario is quite complex, as combined homozygous MICU1 and EMRE deletions causes complete mice lethality (32). Finally, liver-specific downregulation of MICU1 causes an impairment in liver regeneration after injury, due to mPTP opening by mitochondrial Ca^2+^ overload. Indeed, similarly to previously reported cell models (39, 41, 45, 46), MICU1 knockdown in hepatocytes lowers the threshold and decreases the cooperativity of mitochondrial Ca^2+^ accumulation (44). Recently, further insights of the importance of MICU1:MCU ratio in determining tissue-specific mitochondrial Ca^2+^ responses were provided. Comparison between liver and heart tissues lead to the conclusion that the tissue specificity of MICU1:MCU ratio directly reflects the amount of MICU1-free channel. This is turn determines tissue-specific decoding of cytosolic [Ca^2+^]. In particular, while in hepatocytes MICU1 is highly expressed, ensuring high threshold and cooperativity, heart mitochondria display low levels of MICU1, which, in turn, determine low threshold and cooperativity necessary for proper heart function. Interestingly, forced expression of MICU1 induced heart mitochondria to acquire a “liver-like” mitochondrial Ca^2+^ pattern, which caused contractile dysfunction (47).

Studies on flies added further insights on the pathophysiological role of MICU1. In *D. melanogaster*, the *CG4495* gene was identified as putative MICU1 homolog based on the high similarity of essential domains. Silencing of MICU1 in dopaminergic neurons caused shortening of life span and impaired climbing ability, the latter worsened with age (48).

### MICU2

The MICU1 paralog MICU2, identified by bioinformatics in the human genome together with MICU3 (which is mainly expressed in brain, and, to a lesser extent, in skeletal muscle) (49), participates to MCU activity regulation. Similarly to MICU1 silencing (18), silencing of MICU2 impairs mitochondrial Ca^2+^ uptake, and depletion of both MICU1 and MICU2 has an additive effect (49). MICU1 and MICU2 expression levels are dependent one to each other. In detail, MICU2 protein expression shows a direct correlation with MICU1 expression levels in HeLa and HEK293T cell lines, while the effect of MICU2 silencing on MICU1 expression are cell-type dependent (49). However, MICU2 protein levels were unaffected in MICU1^−/−^ MEFs and in primary mouse hepatocytes of MICU1 knockdown mice (44). The physical and functional interaction between MICU1 and MICU2 and their coordinate effects on MCU activity were further examined (46). Patron et al. demonstrated that MICU1 and MICU2 form a heterodimer and play opposing effects on mitochondrial Ca^2+^ entry. In particular, MICU1 has a stimulatory role, while MICU2 inhibits Ca^2+^ uptake. Both are regulated by Ca^2+^ binding at their EF-hand domains. At low cytosolic [Ca^2+^], the inhibitory role of MICU2 prevails, while at high cytosolic [Ca^2+^] conformational modifications ensure prompt MICU1-dependent activation of mitochondrial Ca^2+^ uptake (46). This work also confirmed that MICU1 is essential for MICU2 stability in HeLa cells, and thus, in light of this observation, it reassessed previous data based on MICU1 downregulation. In particular, the increase in mitochondrial Ca^2+^ uptake triggered by MICU1 silencing was due to concomitant destabilization of MICU2 protein. Similarly, Kamer and Mootha demonstrated that MICU1 and MICU2 act together in setting the threshold of the Ca^2+^ response, that MICU2 requires MICU1 for binding to the pore and that the Ca^2+^-binding subunits of both proteins are essential for mitochondrial Ca^2+^ uptake (45). In addition, disulfide bonds catalyzed by the oxidoreductase Mia40/CHCHD4 contribute to MICU1/MICU2 dimer formation and to mitochondrial Ca^2+^ uptake (50). However, contrary to MICU1:MCU ratio, which, as described above, determines tissue-specific mitochondrial Ca^2+^ uptake profiles, MICU1:MICU2 and MICU2:MCU relative abundance does not dictate the mitochondrial Ca^2+^ uptake properties of different tissues (47).

### MICU1.1

Specifically in skeletal muscle, MICU1 gene encodes an alternative splice variant, MICU1.1, which differs from MICU1 for a tiny four amino acid sequence encoded by an alternative exon. This short sequence is sufficient to confer MICU1.1 unique properties. Overexpression of MICU1.1 in HeLa cells causes high increases in mitochondrial Ca^2+^ uptake, which exceed those triggered by MICU1. While MICU2 overexpression reduces MICU1-induced mitochondrial Ca^2+^ uptake (46), it does not exert similar effects on MICU1.1. A detailed analysis demonstrated that MICU1.1–MICU2 heterodimer acts as gatekeeper at resting cytosolic [Ca^2+^], but sets the Ca^2+^ threshold for channel activation at a lower [Ca^2+^] compared to that of the MICU1–MICU2 dimer. This effect is due to the one magnitude higher affinity for Ca^2+^ of MICU1.1 compared to MICU1. MICU1.1 ensures prompt mitochondrial Ca^2+^ uptake in skeletal muscle, which is translated in more efficient ATP synthesis required for muscle contraction (51).

### MCUR1

Finally, MCUR1 is an IMM protein which possesses two TM domains. In mammalian cells, MCUR1 acts as a positive modulator of MCU. Downregulation of MCUR1 decreases mitochondrial Ca^2+^ uptake, oxidative phosphorylation, and ATP production, while AMP kinase-dependent pro-survival autophagy is activated (52, 53). However, the negative effect of MCUR1 downregulation on mitochondrial Ca^2+^ uptake and oxidative phosphorylation has been questioned, and it has been attributed to its dramatic effect on the mitochondrial membrane potential. Rather, a role for MCUR1 as cytochrome c oxidase assembly factor was recognized, which would justify the collapse in membrane potential and mitochondrial Ca^2+^ uptake observed upon its suppression (54). The demonstration that MCUR1 actually plays a role in regulating MCU activity and that the effect on membrane potential is negligible, was provided by recording MCU-mediated Ca^2+^ currents by patch clamp electrophysiology of mitoplasts derived from cells in which MCUR1 was silenced. In experiments in which membrane potential was constantly monitored, stable knockdown of MCUR1 diminished MCU Ca^2+^ currents by 65%, while membrane potential was unaffected (55). In addition, both cardiac and endothelial mitochondria of tissue-specific MCUR1^−/−^ mice showed minimal mitochondrial Ca^2+^ uptake upon extramitochondrial Ca^2+^ pulses despite constant Δψ (53). Ectopic MCUR1 co-immunoprecipitates with MCU (52) and EMRE and functions as scaffold factor required for proper complex assembly (53). However, the 480 kDa complex obtained upon digitonin permeabilization and native gel electrophoresis of MCU-Flag overexpressing HEK293 cells, contained all known MCU regulators with the exception of MCUR1 (56). Finally, MCUR1 plays species-specific functions, since in *D. melanogaster* it controls the Ca^2+^ threshold for permeability transition, independently of its effects on Ca^2+^ uptake rates (57). The intricated results of these studies are likely due to cell type-specific processes, and/or to methodological differences and surely deserve further investigation.

### MCU Activity Modulation

Besides the role of the abovementioned modulatory proteins, additional mechanisms control the channel activity, both transcriptionally and post-transcriptionally. Transcriptional regulation of MCU complex components is far from being completely elucidated; however, some important insights are already available. MCU transcription is controlled by the transcription factor CREB. Specifically, CREB binds to Mcu promoter, and this binding is regulated by cytosolic Ca^2+^. Accordingly, dysregulation of Ca^2+^ homeostasis, triggered by lack of expression of either InsP3R or Orai1 or STIM1, determines lower MCU expression and consequently altered mitochondria metabolism (58). In one study, in which the contribution of MCU to excitotoxicity was assessed, it was also shown that Mcu is subject to transcriptional repression by neuroprotective Ca^2+^ signals. The regulation mechanism requires the induction of immediate-early gene Npas4, which is dependent on nuclear Ca^2+^ and CaM kinase. MCU overexpression exacerbates excitotoxic cell death, while MCU silencing is protective. These findings suggest that MCU activity in neurons is regulated to prevent mitochondrial Ca^2+^-overload and subsequent cell death (59).

Post-transcriptionally, Mcu was reported to be a target of miR-25. In colon cancer, expression of miR-25 and Mcu are inversely correlated. Overexpression of miR-25, by downregulating Mcu, reduces mitochondrial Ca^2+^ uptake and protects cells from apoptotic stimuli. *Vice versa*, anti-miR-25 expression increases mitochondrial Ca^2+^ uptake and sensitizes cells to apopotic death (60). Similarly, miR-25 targets MCU in cardiomyocytes, presumably as a protective effect against oxidative damage (61). Together with miR-25, a second miRNA, i.e., miR-138, was reported to target MCU in pulmonary artery smooth muscle cells (PASMCs). MiR–MCU axis would play a regulatory role in pulmonary arterial hypertension (PAH) by controlling the rate of PASMC proliferation, migration, and sensitivity to apoptosis (62).

Post-translational modifications have also been described. MCU has been proposed to be a target of Ca^2+^/calmodulin-dependent protein kinase II (CamKII). CamKII would positively regulate MCU activity, and CamKII inhibition protects cardiomyocytes from I/R injury (63, 64). However, the role of CamKII in regulating MCU currents has been questioned (65). A second post-translational modification of MCU was observed to be mediated by proline-rich tyrosine kinase 2 (Pyk2) which, in cardiac cells, translocates from the cytosol to the mitochondrial matrix by activation of α_1_-adrenoceptor. Within the mitochondria, Pyk2 phosphorylates MCU, thus stimulating MCU oligomerization and activity (66).

Mitochondrial calcium uniporter is also regulated by mitochondria ROS production. In particular, Cys-97 in the MCU sequence has been identified as a target of mROS and undergoes S-glutathionylation. This modification promotes high-order MCU oligomerization and persistent MCU activity, which in turn triggers mitochondrial Ca^2+^ overload and eventually cell death (67).

Finally, MICU1 appears to be target of protein arginine methyl transferase 1. Accordingly, methylation of MICU1 would represent an additional mechanism of mitochondrial Ca^2+^ uptake regulation (68).

## MCU and Cancer

The idea that mitochondrial Ca^2+^ signaling regulates crucial hallmarks of cancer finds its roots in seminal studies investigating cell functions regulated by mitochondrial Ca^2+^ uptake. In particular, one of the first observations was that mitochondrial Ca^2+^ overload sensitizes cells to apoptotic stimuli, and this has been always viewed as a promising strategy to eliminate aberrant cells, including cancer cells, which would have otherwise escaped apoptotic death (3). In recent years, the role of mitochondrial Ca^2+^ uptake has evolved in parallel to novel hallmark of cancer (69), including the renewed interest for the deregulation of cellular energetics. On this topic, one of the conundrums that continue to puzzle researchers in the field is related to the so-called Warburg effect, i.e., the fact that cancer cells mainly rely on glycolysis-produced ATP despite normal pO_2_ and despite being provided with functional mitochondria, in principle capable of efficient oxidative phosphorylation. The matter is even more intricate if one considers the fact that cancer cell lines (e.g., HeLa, MDA-MB-231, and others) promptly respond to InsP3-generating stimuli with high mitochondrial Ca^2+^ peaks. Clearly, efficient mitochondrial Ca^2+^ uptake in these cells is not primarily utilized to stimulate TCA cycle and oxidative phosphorylation and should have other functions, if any. Partial answers derive from studies of mitochondria metabolism that have unraveled important roles for mROS production, regulation of NADH/NADPH, and utilization of metabolic intermediates in cancer.

To verify whether correlations between MCU complex components expression, tumor progression, and prognosis exist, different approaches have been used. In a study by Hall et al., the Breastmark algorithm, which integrates many DNA microarray experiments for which clinical data are available, was interrogated to determine the relationship between expression of MCU and MICU1 and survival (70). The results of this study indicate that combined overexpression of MCU and underexpression of MICU1 is a feature of patients with poor prognosis, while overexpression of MICU1 and of MCUb, alone or in combination, correlates with better prognosis. A similar analysis was performed by interrogating the Oncomine microarray database bank. In this case, MCU expression was analyzed in different breast cancer subtypes in comparison to normal tissue. The analysis revealed that MCU is overexpressed in breast cancer and also that invasive ductal breast cancer expresses more MCU compared to ductal carcinoma *in situ*. Finally, MCU expression correlates with lymph node invasion (71). When different breast cancer subtypes where compared, a higher expression of MCU was highlighted in estrogen receptor-negative patient samples, especially in basal-like breast cancers compared to luminal A and B subtypes (72). A fourth study correlated mRNA levels of MCU and related proteins (MCUb, MICU1–3, and EMRE) from the TCGA breast cancer dataset with clinical stages (73) demonstrating that MCU expression increases with tumor size and lymph node infiltration, while MCUb expression decreases. Expression of related proteins, i.e., MICU1, MICU2, MICU3, and EMRE did not show significative correlation with tumor stages. In head and neck squamous cell carcinoma, MICU1 expression correlates with the expression of EZH2, which is a negative prognostic factor. EZH2 as well as MICU1 inhibition triggers cell cycle arrest and apoptosis. The EZH2 chemical inhibitor DZNep reduced MICU1 protein levels, suggesting that MICU1 could be involved in EZH2-regulated cell-death pathway (74). Finally, in pancreatic cancer MCU and MICU1 genes undergo loss of heterozygosity (75), although whether MCU and MICU1 are oncogenes or tumor suppressor genes still needs to be clarified. Further studies are required to clarify the expression profile of MCU components in a wider array of cancers.

The metabolic and biological effects of MCU complex modulation have been investigated in different cancers, although the heterogeneity intrinsic of tumor development suggests that much still needs to be explored. In addition, analyses of the expression profiles of the uniporter components in cancer specimens have unravel additional information on the role of MCU on cancer progression.

One of the first reports investigating the role of MCU in cancer progression identified miR-25 as a cancer-related MCU-targeting miRNA family. In detail, in colon adenocarcinoma, miR-25 is overexpressed and, accordingly, MCU expression is inhibited. In agreement with the role of mitochondrial Ca^2+^ uptake in controlling cell survival and death, both in prostate and in colon carcinoma cells, miR-25 overexpression reduces mitochondrial Ca^2+^ uptake and sensitization to apoptotic stimuli, while expression of an “antagomiR” against miR-25 had a deleterious effect on cells that where stressed with an apoptotic challenge (60).

Celastrol, a triterpene extracted from the plant *Tripterygium wilfordii*, was reported to induce mitochondrial Ca^2+^ uptake and ER stress in cancer cells. Celastrol treatment induced paraptosis of these cells, a form of programmed cell-death morphologically and biochemically distinct from apoptosis (76). Intriguingly, MCU silencing partially decreased celastrol-induced cancer cell death, supporting the view of a deleterious effect of Ca^2+^ uptake in stress conditions.

Besides resistance to apoptosis, a fundamental step toward tumor initiation and progression is escape from senescence, a stable proliferation arrest status that exerts a protective function against many pathologies, including cancer. MCU was identified, together with ITPR2, as a novel regulator of both replicative and oncogene-induced senescence (OIS) (77). During OIS, ITPR2 activity triggers Ca^2+^ release from the ER, which is followed by mitochondrial Ca^2+^ accumulation, loss of membrane potential, increased ROS production, and senescence. *Vice versa*, loss of MCU enables escape from OIS.

In a particular cancer model, i.e., triple negative breast cancer (TNBC), the role of MCU has been extensively investigated both in terms of cell viability and on migration/invasion properties (Table 1). Based on the expression data reported above, suggesting increased MCU expression in basal-like breast cancer, Curry et al. hypothesized a survival advantage triggered by MCU (72). Accordingly, MCU was silenced in MDA-MB-231 cell line and cell viability and resistance to stress stimuli was measured. MCU silencing *per se* did not affect at all cell proliferation and viability. Also, when cells were treated with the BCL-2 inhibitor ABT-263, caspase-dependent cell death was unaffected. However, caspase-independent death triggered by treatment with the Ca^2+^ ionophore ionomycin was potentiated by MCU knockdown, suggesting that MCU inhibition could in principle be viewed as a therapeutical strategy against TNBC.

**Table 1:** Summary table on the role of MCU in triple negative breast cancer (TNBC).

Breast cancer patients: expression data vs prognosis	Biological effects of uniporter modulation in TNBC	Reference
MCU high in ER-negative samples and in basal-like breast cancers	MCU silencing: no effect on TNBC cell viability Increased caspase-independent cell death	Curry et al. (72)
MCU high and MICU1 low = poor prognosis MICU1 high and MCUB high = better prognosis	MCU and MICU1 silencing does not affect survival of TNBC cells exposed to irradiation, chemotherapeutic agents, or nutrient deprivation MCU overexpression does not affect ceramide-induced toxicity	Hall et al. (70)
MCU high in breast cancer MCU higher in invasive ductal breast cancer than in ductal carcinoma *in situ* MCU high in patients with lymph node invasion	MCU silencing impairs store-operated Ca^2+^ entry (SOCE), which is essential for TNBC cell migration	Tang et al. (71)
Expression of MCU increases and expression of MCUb decreases with breast cancer progression and increased lymph node infiltration	MCU silencing impairs cell migration, ROS production, and HIF1alpha expression in TNCB cells, as well as *in vivo* tumor growth and progression The effects of MCU silencing on SOCE are cell-type dependent	Tosatto et al. (73)

A similar hypothesis was tested by Hall et al. (70). As reported above, their expression study convincingly correlated combined overexpression of MCU and underexpression of MICU1 with poor patient survival. Thus, MCU and MICU1 were silenced in MDA-MB-231 cells, and the effects on survival of cells exposed to irradiation, chemotherapeutic agents, or nutrient deprivation were measured. However, while cervical cancer HeLa cells and non-cancerous breast epithelial HMEC cells showed reduced survival, MDA-MB-231 cells were insensitive to changes in MCU activity. In addition, also overexpression of MCU was ineffective in altering cell response to ceramide-induced toxicity. Thus, the role of mitochondrial Ca^2+^ uptake in cancer cell survival depends on cancer type and on stress stimuli, not to mention the role that cancer niche and extracellular environment exert. In this context, the role of MCU is still obscure.

A different issue is the role that MCU plays in modulating motility and invasion potential of cancer cells. Two reports agreed in indicating MCU as an essential modulator of MDA-MB-231 cell migration (71, 73); however, different mechanisms were proposed. According to Tang et al., MCU silencing inhibited serum- or thapsigargin-induced store-operated Ca^2+^ entry (SOCE) which, in turn, was required for proper cell migration. Tosatto et al. confirmed the data that MCU is required for MDA-MB-231 migration and extended this observation to other triple-negative breast cancer cell lines. However, despite the consistent role on cell motility, the effect of MCU silencing on SOCE was cell line dependent, indicating that different mechanisms are involved, likely directly dependent on the strong reduction in mitochondrial Ca^2+^ uptake, rather than on a secondary effect on global Ca^2+^ homeostasis. MCU inhibition caused a strong reduction in mROS production and HIF-1α expression. In addition, restoring HIF-1α levels was sufficient to overcome the decrease in motility. The essential role of MCU in TNBC progression was demonstrated by *in vivo* experiments in which MDA-MB-231 cells were injected into the fat pad of immune-deficient SCID mice. Deletion of MCU markedly retarded primary tumor formation. In addition, at equal primary tumor size, absence of MCU impaired lung and lymph node colonization (73).

These studies indicate that the MCU complex plays different roles in different cancer types and stages. Research is still needed to extend the analysis to *in vivo* models and to additional cancers and to clarify whether mitochondrial Ca^2+^ uptake impinges on the tumor microenvironment.

## MCU in Other Pathophysiological Settings

**Figure 2:**
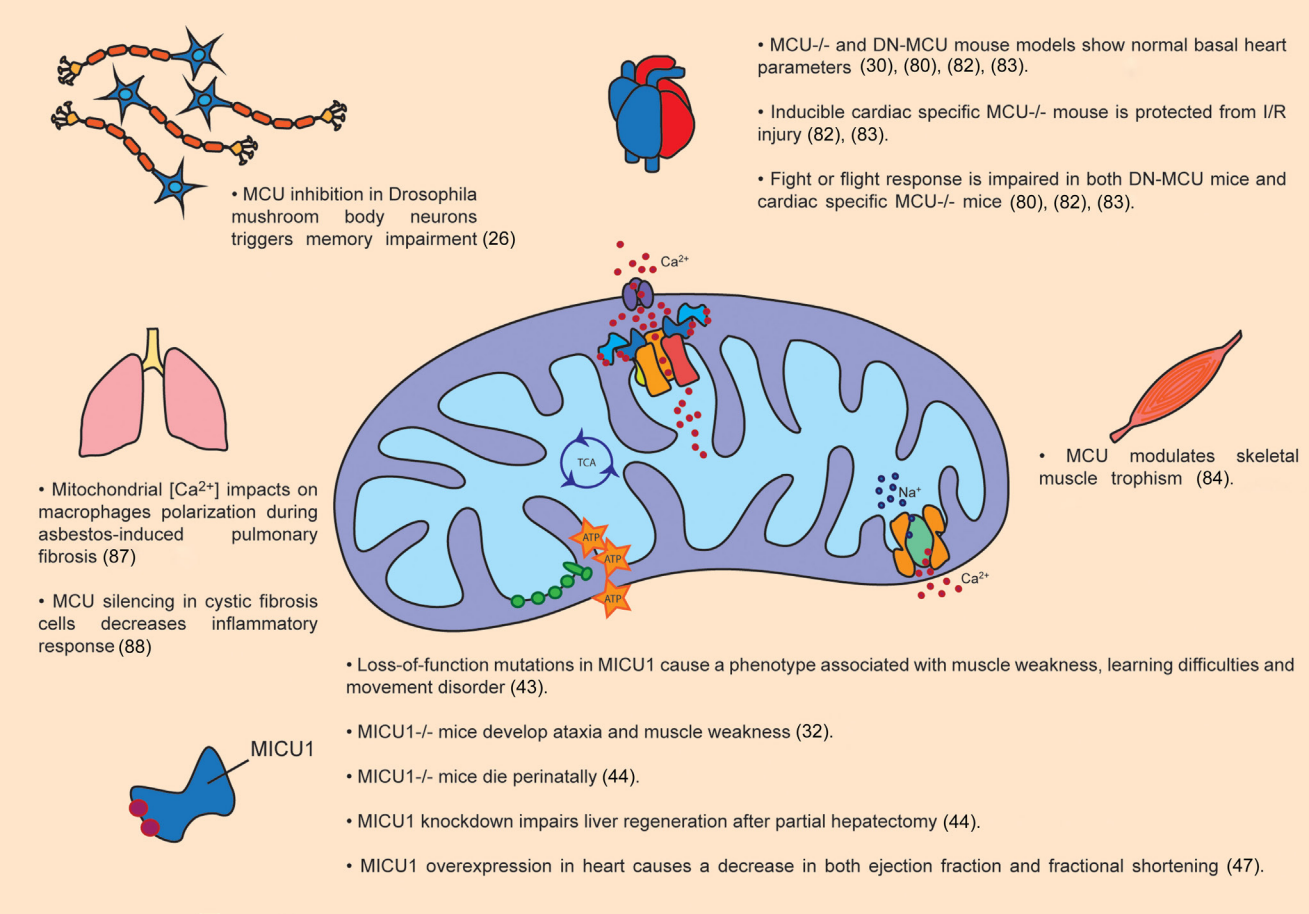
Pathophysiological roles of MCU complex. The figure summarizes the main effects of MCU activity modulation in organs and tissues, both in animal models as well as in patients.

An important amount of work has been done to disclose the pathophysiological role of MCU in different tissues (Figure 2).

On this issue, one of the first studies after the identification of MCU was carried out in neonatal cardiomyocytes *ex vivo*, demonstrating that MCU expression regulates the buffering of cytoplasmic Ca^2+^ during systole. In detail, cytoplasmic Ca^2+^ peaks are increased or reduced by MCU silencing or overexpression, respectively (78). However, the first *in vivo* analysis of MCU^−/−^ hearts gave unexpected results that are still debated. MCU^−/−^ hearts show no sign of protection against I/R injury (30). Interestingly, while wild-type hearts were protected from I/R injury when treated with CsA, which inhibits Ca^2+^-dependent cell death mediated by the opening of mPTP, MCU^−/−^ hearts lacked CsA protection. This result suggests that a Ca^2+^-independent death pathway occurs in the absence of MCU. Experiments on isolated heart mitochondria showed impaired mitochondrial Ca^2+^ accumulation and reduced matrix Ca^2+^ levels in MCU^−/−^ strain (79), consistent with the data previously reported on skeletal muscle isolated mitochondria (30). However, basal ATP levels were similar between MCU^−/−^ and wild-type hearts, suggestive of preserved basal metabolic function. In addition, an accurate study of heart morphology by echocardiographic analysis of 12- and 20-month-old mice revealed similar stroke volume, ventricular volumes, and wall thickness between MCU^−/−^ and wild-type controls, indicating that MCU^−/−^ mice maintain basal cardiac function over at least 20 months of life (79). Upon physiological stress induced by isoproterenol stimulation, left ventricular cardiac output and heart rate were undistinguishable from those of wild-type controls even though MCU^−/−^ cardiomyocytes did not accumulate Ca^2+^. Also upon chronic stress, induced by transverse aortic constriction (TAC), MCU^−/−^ and wild-type genotypes display similar decline in ejection fraction, fractional shortening, hypertrophy and fibrosis.

Additional studies were carried out in a transgenic mouse model, expressing a DN-MCU (80, 81). As expected, the heart mitochondria from DN-MCU mice showed loss of mitochondrial Ca^2+^ accumulation (80), although DN-MCU does not completely abolish mitochondrial Ca^2+^ uptake when expressed in HeLa cells (16). DN-MCU mice showed hearts with normal chamber size and heart beat. Increased heart rate triggered by isoproterenol treatment requires enhanced ATP production necessary to support SERCA activity and appropriate Ca^2+^ load of the sarcoplasmic reticulum in the SAN (sinus atrial node) cells. MCU inhibition impaired fight or flight heart rate by decreasing ATP levels, and dialysis of ATP was sufficient to rescue the phenotype in cardiac pacemaking cells (80). Enhanced oxygen consumption was a peculiar feature of DN-MCU perfused hearts, while it was not observed in isolated mitochondria or permeabilized cardiomyocytes of the same animals, suggesting that the increased energetic demand is related to extramitochondrial Ca^2+^ homeostasis. In addition, DN-MCU ventricular cardiomyocytes exhibited increased cytosolic [Ca^2+^], in line with the Ca^2+^ buffering effect of mitochondria, which was partially overturned by ATP dialysis (81). Consistent with results obtained in MCU^−/−^ mouse (30), DN-MCU expression was not protective against myocardial death induced by I/R injury, despite maintenance of ΔΨm and decreased ROS production (81).

In both mouse models discussed above, embryonic adaptations to MCU modulation could not be excluded. To overcome this issue, in 2015 a new mouse model was generated, with two LoxP sites flanking exons 5 and 6 of MCU gene. These animals were crossed with mice expressing a tamoxifen-inducible Cre recombinase driven by a cardiomyocyte-specific promoter (α-myosin heavy chain promoter). MCU gene deletion was induced in adult mice, and cardiac function was evaluated (82, 83). Similarly to DN-MCU and MCU total knockout mice, basal cardiac function and heart morphology were normal. On the other hand, studies of inducible cardiac-specific MCU^−/−^ mice confirmed the impairment in the fight or flight response. Cardiomyocytes derived from these mice had normal respiration rate in basal conditions, while decreased oxygen consumption rate after β-adrenergic stimulation suggesting a MCU pivotal role during increase heart workload. However, no differences were observed in fractional shortening, cardiomyocytes area and heart weight upon TAC. Finally, as opposed to constitutive MCU^−/−^ mice and DN-MCU animals, inducible MCU ablation strongly protected hearts from I/R-induced cell death.

Besides heart, another tissue strictly dependent on oxidative metabolism is skeletal muscle. Skeletal muscle is the most affected tissue in constitutive MCU^−/−^ mice although at very mild levels. Skeletal muscle mitochondrial matrix [Ca^2+^] was reduced compared to wild-type controls, although not completely abolished. A decrease maximal oxygen consumption rate was detected. In addition, PDH activity was impaired in agreement with the pivotal role of mitochondrial Ca^2+^ accumulation in the control of ATP production. Finally, MCU^−/−^ animals show defective maximal power output generation. Subsequently, to discriminate the compensatory effects occurring during embryonic development, MCU modulation was achieved after birth by means of adeno-associated viral vectors for both overexpression and silencing (84). Modulation of mitochondrial Ca^2+^ accumulation was shown to contribute to skeletal muscle trophism. Indeed, MCU overexpression and downregulation trigger muscle hypertrophy and atrophy, respectively, both during postnatal development and in adulthood. Most importantly, MCU overexpression protects from denervation-induced muscle atrophy caused by sciatic nerve excision suggesting a possible therapeutic role of mitochondrial Ca^2+^ uptake in muscle atrophy. Surprisingly, these effects are independent from the control of aerobic metabolism as demonstrated by different evidence. First, PDH activity, although defective in MCU silenced muscles, was unaffected in MCU overexpressing muscles. Second, hypertrophy was comparable in both oxidative and glycolytic muscles and, finally, analyses of aerobic metabolism revealed no major alterations. Conversely, the control of skeletal muscle mass by mitochondrial Ca^2+^ modulation is due to the role of two major hypertrophic pathways of skeletal muscle, PGC-1α4 and IGF1-AKT/PKB. Taken together, these results demonstrate the existence of a Ca^2+^-dependent mitochondria-to-nucleus signaling route that clearly links organelle physiology to the control of muscle mass. As mentioned above, loss-of function mutations in MICU1 genes cause a disease which includes neuromuscular defects and learning difficulties (43), and MICU1 deletion in mice is either lethal (44) or causing similar phenotype to the human disease (32). Finally, a recent work carried out by Zampieri and coworkers reported increased skeletal muscle MCU protein levels in human seniors subjected to muscle training, suggesting that MCU represents a potential pharmacological target to counteract sarcopenia (85).

Beside striated muscles, the role of MCU has been studied also in other organs in which mitochondria play a crucial role for tissue-specific functions. One case is represented by endocrine pancreas, specifically β-cells. Soon after MCU discovery, experiments carried on in pancreatic β-cells demonstrated that MCU-mediated mitochondrial Ca^2+^ uptake is essential for cell depolarization-induced increase in ATP/ADP ratio (86). In addition, culture conditions mimicking diabetic milieu delayed mitochondrial Ca^2+^ uptake. Thus, MCU is an essential component of glucose sensing by pancreatic β-cells.

In a totally different context, a recent article reported evidence that mitochondrial [Ca^2+^] could have an impact on macrophage polarization (87). Gu and coworkers demonstrated that MCU^+/-^ mice are protected from asbestos-induced pulmonary fibrosis. In detail, macrophages of MCU^+/-^ mice showed attenuated profibrotic polarization after asbestos exposure together with a decrease in ATP production. In addition, histological analysis revealed normal lung architecture without collagen deposition in MCU^+/-^ mice. These results suggest that the modulation of mitochondrial Ca^2+^ accumulation could be a promising therapeutic target for human fibrosis.

Additional demonstration of the pivotal role of mitochondrial Ca^2+^ uptake in pathophysiology is described by Rimessi and coworkers (88). This study analyzes the *Pseudomonas aeruginosa*-induced inflammatory process that takes place in cystic fibrosis cells. When inflammation is triggered in a cystic fibrosis cell line model, MCU silencing causes a decrease in inflammasome activation, indicating that mitochondrial Ca^2+^ accumulation is a potential therapeutic target.

Finally, MCU is a target of miR-25, and miRNA-dependent MCU modulation has consequences on different physiopathological settings, as detailed in the Sections “MCU Activity Modulation” and “MCU and Cancer” (60, 61). Also miR-138 has been reported to play a regulatory role on MCU in the context of PAH (62).

## Conclusion

The seminal discoveries of *cbara1* as the gene encoding MICU1, and of *ccdc109a* as the one encoding MCU, followed by the identification of the other components of the uniporter, determined the possibility of conducting genetic and molecular studies on mitochondrial Ca^2+^ uptake mechanism and significance. In a simplified view, so far research has progressed in different directions to define: (a) the identity and role of uniporter channel components and interacting subunits, and their mutual regulation; (b) the transcriptional and posttranscriptional mechanisms that contribute to channel activity control; and (c) the pathophysiological consequences of dysregulated mitochondrial Ca^2+^ signaling. For any of these topics, impressive progresses have been achieved in a relatively short time. However, many questions are still unanswered. First, work is still needed to better clarify how the different channel subunits and regulatory components functionally interact with each other and to determine cell-type specificity of these mechanisms. Second, the regulation of uniporter components expression and activity by transcriptional and posttranscriptional events is a still largely unexplored field. Third, the pathophysiological role of MCU in different tissues is still unclear. Specifically on tumor development and progression, the role and significance of mitochondrial Ca^2+^ signaling has been studied only in few cancer types, and nothing is known on the effects of MCU activity in the tumor niche. In addition, although patient-derived samples have been analyzed for the expression levels of MCU components and correlation data between expression and prognosis have been obtained, *in vivo* tumorigenesis experiments aimed to determine the significance of aberrant mitochondrial Ca^2+^ uptake at different cancer stages have been rarely performed. Research in the next years will uncover these and others aspects of mitochondrial Ca^2+^ signaling.

## Author Contributions

CM, GG, and RR wrote the manuscript. GG prepared figures.

## Conflict of Interest Statement

The authors declare that the research was conducted in the absence of any commercial or financial relationships that could be construed as a potential conflict of interest.
